# Psychological distress in Portuguese university students during COVID-19 pandemic: relationship with stress, sleeping and emotion regulation strategies

**DOI:** 10.1192/j.eurpsy.2023.1655

**Published:** 2023-07-19

**Authors:** J. Sousa, M. J. Soares, D. Pereira, A. Macedo

**Affiliations:** 1 Department of Psychological Medicine, Faculty of Medicine, University of Coimbra; 2 Department of Psychiatry, Centro Hospitalar e Universitário de Coimbra, Coimbra, Portugal

## Abstract

**Introduction:**

During the COVID-19 pandemic outbreak, psychological distress, anxiety and depression reached new highs associated with a number of variables such as pandemic related-stress and sleep difficulties. These later two are known to be the precipitant and risk factors for psychological distress/mental disorders, respectively, and negative cognitive emotion regulation strategies can also have a key role on psychological/mental health problems generation and maintenance.

**Objectives:**

To study stress, sleep difficulties and the use of cognitive emotion regulation strategies by groups of students with different levels of psychological distress during the COVID-19 pandemic.

**Methods:**

496 university students (mean age ± SD=20.99 years ± 2.27; 78.6% women) completed an online questionnaire between January and April, 2021, which included the Mental Health Inventory, the Perceived Stress Scale (PSS) and the Cognitive Emotion Regulation Questionnaire, as well as three questions from the Insomnia Scale to evaluate Difficulties in Initiating Sleep (DIS), Maintaining Sleep (DMS) and Early Morning Awakening (EMA). A Sleep Difficulties Index (SDI) was calculated by summing the scores of these three items.

**Results:**

18.3% of the students showed high levels of psychological distress (group 1; scores 1 SD =/< Mean), 62.7% average levels (group 2), and 19% low levels (group 3; Scores 1SD =/>Mean). The group 1, compared with group 3, showed higher levels of sleeping difficulties (DIS, DMS, EMA and SDI), greater stress levels and an increased use of negative emotion regulation strategies, as well as a lesser use of positive emotion regulation strategies. Furthermore, the group 2, with average levels of psychological distress is significantly distinct from the group with better mental health when comparing these same variables.

**Conclusions:**

Almost one fifth of the Portuguese university students report elevated levels of psychological distress during the COVID-19 pandemic. Clinical interventions to promote psychological/mental health in this population should focus on reducing stress, promoting adequate sleep habits, reducing the use of negative cognitive emotion regulation strategies and increasing the use of positive ones.

**Disclosure of Interest:**

None Declared

